# Association study of functional genetic variants of innate immunity related genes in celiac disease

**DOI:** 10.1186/1471-2350-6-29

**Published:** 2005-08-03

**Authors:** B Rueda, A Zhernakova, MA López-Nevot, J Martín, BPC Koeleman

**Affiliations:** 1 Instituto de Parasitología y Biomedicina "López-Neyra", CSIC, Granada, Spain; 2 Complex Genetics Group, Department of Biomedical Genetics, University Medical Center, Utrecht, The Netherlands; 3 Servicio de Inmunología, Hospital Virgen de las Nieves, Granada, Spain

## Abstract

**Background:**

Recent evidence suggest that the innate immune system is implicated in the early events of celiac disease (CD) pathogenesis. In this work for the first time we have assessed the relevance of different proinflammatory mediators typically related to innate immunity in CD predisposition.

**Methods:**

We performed a familial study in which 105 celiac families characterized by the presence of an affected child with CD were genotyped for functional polymorphisms located at regulatory regions of *IL-1α, IL-1β, IL-1RN, IL-18, RANTES *and *MCP-1 *genes. Familial data was analysed with a transmission disequilibrium test (TDT) that revealed no statistically significant differences in the transmission pattern of the different genetic markers considered.

**Results:**

The TDT analysis for *IL-1α, IL-1β, IL-1RN, IL-18*, and *MCP-1 *genes genetic variants did not reveal biased transmission to the affected offspring. Only a borderline association of *RANTES *promoter genetic variants with CD predisposition was observed.

**Conclusion:**

Our results suggest that the analysed polymorphisms of *IL-1α, IL-1β, IL-1RN, IL-18, RANTES *and *MCP-1 *genes do not seem to play a major role in CD genetic predisposition in our population.

## Background

Celiac disease (CD) is an autoimmune disorder of the small intestine in which dietary gluten ingestion leads to a chronic inflammatory status of the mucosa [1]. There is strong evidence for a genetic component for CD, with the HLA genes being the strongest genetic locus associated with CD predisposition known to date. About 95% of CD patients are carriers of the DQ2 molecule, encoded by DQA1*05/DQB1*02 alleles, compared to ~10% of healthy control subjects. Furthermore, the DQ8 molecule (DQA1*0301/DQB1*0302 is also found more frequently in CD patients although to a lesser extent [2]. Finally, a role for genes located outside the HLA region has been suggested since the overall contribution of HLA genes to CD genetic predisposition is no more than 40% [1].

T CD4+ lymphocytes are key elements in the induction and progression of CD pathogenesis. Certain gluten peptides bound to DQ2 or DQ8 molecules cause proliferation and production of proinflammatory cytokines by lamina propria CD4 +T cells [3]. Besides this activation of adaptive immune response, recent evidences suggest that there is an implication of the innate immunity in the initial phases of CD [4]. In this regard, some gluten peptides have been demonstrated to drive a danger signal that leads to an activation of the innate immune system [5,6] and additionally it is thought that bacteria may play a role in CD [7]. In fact, CD patients show an up-regulation in the expression of pro-inflammatory cytokines typically related to the innate immune response, such us IL1, IL-18 and chemokines [6,8-10].

The IL1 gene cluster located in the chromosomal region 2q12-22 codifies for three proteins: IL-1α, IL-1β and IL-1 receptor agonist (IL-1RN), of which the two first are strong inducers of inflammation while IL-1RN is an effective antagonist binding to the IL-1 receptor without activating the target cell [11]. These genes are polymorphic bearing well-characterized single nucleotide polymorphisms (SNPs). Polymorphisms in IL-1α at position -889 C/T (rs1800587) and IL-1β at position -511 C/T (rs1143627) were described [12,13]. Furthermore, recent findings showed that the -511 C/T IL-1β genetic variant is related to differences in IL-1β protein secretion [14]. The *IL-1RN *gene contains within its second intron a variable number of an 86-bp tandem repeats (rs380092) [15], showing the allele 2 (IL-1RN*2; two repeats) an increased frequency in a variety of autoimmune and inflammatory disorders [16].

Another important member of the proinflammatory IL-1 family is IL-18, which is thought to be a key regulator of cytokine expression [17]. Furthermore, a role for IL-18 in the induction of an anti-gluten inflammatory response has been suggested [10,18,19]. It is thought that IL-18 gene variation in the promoter region regulates the expression of this cytokine [20]. Interestingly, in the IL-18 promoter region two SNPs -607 A/C (rs1946518) and -137 G/C (rs187238) were described, which are supposed to alter the IL-18 promoter activity [21].

Moreover, raised levels of chemokines such us RANTES (regulated upon activation, normal T-cells expressed and secreted) and monocyte chemoatractant protein-1 (MCP-1) have been observed in the primary immune response to gluten in CD patients [6,8]. Interestingly, genetic variants within regulatory regions that can affect trancription and protein production levels, *RANTES *-403 G/A (rs2107538) and -28 G/C (rs2280788) and *MCP-1 *-2518 G/A (rs1024611) SNPs, were described [22-24].

Taking into consideration these findings, in this work we aimed to investigate the possible implication of *IL-1α, IL-1β, IL-1RN, IL-18, RANTES *and *MCP-1 *functional polymorphisms in CD susceptibility.

## Methods

### Patients

In the present work we have analysed a panel of 105 celiac families characterised by the presence of an affected child with CD. The study participants were recruited at "Hospital Materno-Infantil" and "Hospital Clinico Universitario", Granda, (Spain) and were of Spanish Caucasian origin. All patients were diagnosed following the European Society of Paediatric Gastroenterology, Hepatology and Nutrition (ESPGHAN) criteria for CD [25]. Their age at study was 7.1 ± 3.9 years and the mean age for disease diagnosis was 2.7 ± 2.72. A 60% were women and 40 % men, showing an anthropometry at diagnosis (weight and height) of P 3–100 percentile. The mean age of gluten introduction was 6.4 ± 1.5 months. Typical symptoms were observed in 72.2 % of patients and 27.8 % showed atypical symptoms. All family members were genotyped in DRB1 and DQB1. DQA1 typing was deduced from DQB1 and DRB1 typing on the basis of the strong linkage disequilibrium among HLA class II alleles.

### Genotyping

DNA from patients and controls was obtained from peripheral blood using standard methods. For all of the considered SNPs, except *IL-1RN *and *RANTES *-403, samples were genotyped using a Taqman 5' allelic discrimination assay. Table 1 shows the Taqman MGB probes sequences used for each polymorphism provided by the Custom-Taqman-SNP-Genotyping-Assay (Applied Biosystems, Foster City, CA, USA). PCR reaction was carried in a total reaction volume of 5 μl with the following amplification protocol: denaturation at 92°C for 10 min, followed by 50 cycles of denaturation at 92°C for 15 sec and annealing and extension at 58°C for 1 min. Post-PCR, the genotype of each sample was attributed automatically by measuring the allelic specific fluorescence on the ABI PRIM 7900 Sequence Detection Systems using the SDS 2.2.1 software for allelic discrimination (Applied Biosystems, Foster City, CA, USA). *RANTE*S -403 genotyping was performed using a TaqMan SNP-Genotyping-Assay (part number: C__15874407_10, Applied Biosystems, Foster City, CA, USA).

The *IL-1RN *polymorphism was genotyped by PCR as previously described [26]. Briefly, we used a froward primer 5'- CTC AGC AAC ACCT CCT AT and reverse primer 5'- TCC TGG TCT GCA GGT AA, two amplify five possible alleles with different PCR fragment size: 410 bp (allele 1: 4 repeats), 240 bp (allele 2: two repeats), 325 bp (allele 3: 3 repeats), 500 bp (allele 4: 5 repeats), and 595 bp (allele 5: 6 repeats).

### Statistical analysis

We used the UNPHASED software created for TDT and case-control analysis [27]. We performed a Transmission Disequilibrium Test (TDT), which assesses allele transmission rates in simplex families and tests for deviation from expected 50% transmission. For the haplotype analysis, pair-wise linkage disequilibrium measures were investigated and haplotypes constructed using the expectation-maximization (EM) algorithm implemented in UNPHASED software. The power of the study to detect an effect of a polymorphism in disease susceptibility was estimated using the Quanto v 0.5 software (Department of Preventive Medicine University of Southern California, California, USA) [28].

## Results

### IL1 gene cluster

The transmission pattern for *IL-1α *-889, *IL-1*β -511 and *IL-1RN *VNTR polymorphisms is shown in table 2. When transmission of these genetic variants was analysed, none of the alleles showed statistically significant skewing. IL-1α -889 T allele was slightly more transmitted to the affected children (58% transmission for allele T vs 47% for allele C), however the *p *value failed to reach statistically significant level (Table 2). With regard to *IL-1RN *we observed that alleles IL-1RN*1 and IL-1RN*2 were the most frequent in our population (71.6% and 26.8% respectively), accordingly with previously studies in Caucasian populations [26].

### IL18 gene

The TDT analysis for -607 A/C and -137 G/C *IL-18 *promoter genetic variants did not reveal biased transmission of any of the alleles to the affected offspring (Table 2).

The haplotype estimation for the -607 A/C and -137 *IL-18 *promoter variants revealed complete linkage disequilibrium between the two variants (D' = 1). We observed three out of the four possible haplotypic combinations in CD families (Table 3). The transmission pattern of *IL-18 *promoter haplotypes did not show any statistically significant skewing (Table 3).

### MCP-1 and RANTES

After analyzing the *MCP-1 *-2518 G/A alleles transmission we observed that none of the alleles was preferentially transmitted from heterozygous parents to the affected offspring (Table 2). Regarding to the *RANTES *promoter genetic variants the mutant alleles -403 A and -28 G showed an overall allele frequency similar to that expected for Caucasian populations (84.1% and 96.5% respectively in our population) [29,30]. The transmission of both -403G/A and -28 C/G SNPs showed a slightly deviation from the 50% expected transmission pattern (Table 2). Alleles -403 G and -28 C were more transmitted to the affected offspring with borderline significance (*P *= 0.04 and *P *= 0.06 respectively) (Table 2). In addition, we estimated haplotypes for both genetic variants. Three out of the four haplotypic combinations were observed, being the -403G/-28C and -403A/-28C haplotypes the most common in CD families. No significant distorted transmission pattern for *RANTES *promoter haplotypes was observed (Table 3).

## Discussion

CD is considered a model for autoimmune disorders since many of the components that generate the altered immune response to gluten have been well characterized [1]. However, there are some relevant events of CD pathogenesis that remain unclear, for instance the stimuli that drives the high IFNγ levels in the small intestine of CD patients and why only one out of 20–30 DQ2-positive individuals develops CD [3]. An explanation for these questions might be provided from recent studies that point out a role for the innate immunity in CD [4]. This finding supports a novel focus of research in CD molecular and genetic basis, opening a new field for the functional search of CD candidate genes.

In this work, for the first time we have assessed the relevance of *IL-1α, IL-1β, IL-1RN, IL-18, RANTES *and *MCP-1 *genes in CD predisposition. All these genes have been previously associated with susceptibility to several autoimmune disorders [31-40]. However, we failed to detect an association of *IL-1α, IL-1β, IL-1RN, IL-18*, and *MCP-1 *genes with CD predisposition using a TDT analysis in our cohort of 105 simplex CD families. Only a borderline significant association of *RANTES *promoter genetic variants with CD predisposition was observed.

Several studies have focused on the role of *RANTES *-403G/A and -28 G/C promoter polymorphisms in susceptibility to different autoimmune disorders. The *RANTES *-403A allele has been associated with susceptibility to multiple sclerosis (MS) and polymialgia rheumatica [41,42]. On the other hand, the *RANTES *-28G allele was observed to be a genetic risk for clinical complications such us diabetic neprhopathy, early onset of MS, lower levels of C3 in SLE, and higher incidence of central nervous system lupus [37,38,41]. Both *RANTES *-403A and -28G alleles were associated with higher RANTES expression levels [22,23]. However, considering the multiple testing of the 6 different genes of our study, the association observed for *RANTES *promoter variants in our population can not be considered as being significant. Therefore, our results of *RANTES *suggest that further studies should be performed to clarify the role of *RANTES *in CD and autoimmune diseases in general.

Using a familial approach we eliminate the risk of population stratification derived from case-control association studies. In addition, we estimated that our study design would have considerable power to detect the effect of a polymorphism with moderate to high risk for CD. Assuming an additive model, a minor allele frequency of 0.30 (corresponding to a median value of the majority of markers considered) and RR of 1.8 we would reach 81% power to detect an association in our population. Nevertheless, under a dominant model the power drops to 49% and considering a lower disease allele frequency, for instance 0.16 as is the case of *RANTES *-28, our study power would decrease to a 64% for a RR of 1.8, and increases to 82% when we assume a RR of 2.0. For this reason, the low level of significance that our TDT analysis reached for *RANTES *promoter genetic variants might well reflect a true positive, and therefore needs further confirmation using a larger group of CD families.

Taking into account our findings, it is suggested that the analysed genetic polymorphisms of *IL-1α, IL1-β, IL-1RN, IL-18, RANTES *and *MCP-1 *genes seem not to play a major role in CD susceptibility in our population. It might be possible that the release of these cytokines and chemokines observed in CD patients could be derived from the activity of other innate immunity related pro-inflammatory mediators with higher influence in disease pathogenesis. In this regard, it is known that in CD the cytokine expression pattern in response to gluten is strongly dominated by IFNγ [43]. Of note, in a recent work we assessed the influence of a functional dinucleotide polymorphism of IFNγ gene in CD predisposition. An association of a higher IFNγ producer allele with CD was observed, supporting a possible explanation for the high levels of INFγ observed in intestinal mucosa of CD patients [44].

Other proinflammatory mediators related with innate immunity such as, TNF-α and IL-12, has been analysed with respect to CD susceptibility. In accordance with our findings no evidence of association was found between IL-12 and CD in two independent studies [45,46]. Regarding TNF-α it has been difficult to dissect the relevance of this genetic marker in CD since it maps within HLA clas III region and it shows linkage disequilibrium with CD disease predisposing DQ2 alleles. In fact controversial results have been obtained, and there is no consensus about an independent or due to linkage disequilibrium role of TNF-α in CD susceptibility [47,48].

## Conclusion

Our results suggest that *IL-1α, IL1-β, IL-1RN, IL-18, RANTES *and *MCP-1 *genetic variants do not play a major role in CD genetic predisposition, although the suggestive evidence for *RANTES *deserves further investigation. Furthermore, we consider the innate response an intriguing focus of research and it should be of interest to investigate the role of other cytokines up-regulated in the early events of CD.

## Competing interests

The author(s) declare that they have no competing interests.

## Authors' contributions

B.R., carried out the genotyping and statistical analysis and drafted the manuscript.

A.Z., participated in the genotyping and helped in the use of the ABI PRIM 7900 Sequence Detection Systems and SDS 2.2.1 software.

M.A. L-N., collected the samples and revised the manuscript.

J.M., participated in the manuscript design and coordination and helped to draft the manuscript

B.K., reviewed the statistical analysis and helped to draft the manuscript.

All authors read and approved the final manuscript.

## Pre-publication history

The pre-publication history for this paper can be accessed here:



## Figures and Tables

**Table 1:** Taqman probes used for cytokine genotyping

**Polymorphism**	**Taqman probe sequence**
**IL-1A -889 C/T (rs1800587)**	VIC – CCTTCAATGGTGTTGCC
	FAM – CCTTCAATGATGTTGCC
**IL-1B -551 C/T (rs1143627)**	VIC – CTGTTTTTATGGCTTTCA
	FAM – CTGTTTTTATAGCTTTCA
**IL-18 -607 C/A (rs1946518)**	VIC – ATCATTAGAATTTTATGTAATAAT
	FAM – ATCATTAGAATTTTATTTAATAAT
**IL-18 -137 G/C (rs187238)**	VIC – ACTATTTTCATGAAATCTTTTCT
	FAM – TTTTCATGAAATGTTTTCT
**RANTES -28 G/C (rs2280788)**	VIC – CCCCTCAACTGGC
	FAM – CCCCTGAACTGGC
**MCP-1 -2518 G/A (rs1024611)**	VIC – CAGACAGCTGTCACTTT
	FAM – CAGACAGCTATCACTTT

**Table 2:** Allelic frequencies and percentage of transmission of *IL-1α, IL-1β, IL-1RN, IL-18, RANTES *and *MCP-1 *genetic variants in CD families.

	**Alelle frequency in parents (%)**	***T:NT***	***% T***	***P***
**IL-1A -551**				
**C**	70.4	111:125	47	0.08
**T**	29.6	52:38	58	0.08
**IL-1B -889**				
**T**	67.6	110:111	49.7	NS
**C**	32.4	47:46	50.5	NS
***IL-1RN*** **VNTR**				
1	71.6	107:107	50	NS
2	26.8	43:42	50.6	NS
3	1.6	2:2	50	NS
**IL-18 -607**				
**C**	61.7	102:103	49.7	NS
**A**	38.3	59:58	50.4	NS
**IL-18 -137**				
**G**	75.9	124:124	50	NS
**C**	24.1	47:47	50	NS
**RANTES -28**				
**C**	84.1	155:148	51	0.04
**G**	15.9	3:10	23	0.04
**RANTES -403**				
**G**	85.7	141:128	52	0.06
**A**	14.3	23:36	39	0.06
**MCP-1**				
**A**	72.8	117:129	47	NS
**G**	27.2	48:36	57	NS

*T *= transmitted, *NT *= not transmitted *%T *= percentage transmitted

**Table 3:** Transmission pattern of haplotypes inferred for IL-18 and RANTES promoter genetic variant in CD families

**Gene**	**Haplotype**	**Transmitted (%)**	**No transmitted (%)**	***P***
**Il-18**	-607A/-137G	37 (24)	35 (22.5)	NS
	-607A/-137C	19 (12.2)	20 (13)	NS
	-607C/-137G	99 (63.8)	100 (64.5)	NS
**RANTES**	-403G/-28A	3 (1.4)	8 (5.2)	NS
	-403A/-28C	19 (12.3)	26 (16.8)	NS
	-403G/-28C	132 (85.7)	119 (81.5)	0.05
